# Advancing Precision Medicine: The Role of Genetic Testing and Sequencing Technologies in Identifying Biological Markers for Rare Cancers

**DOI:** 10.1002/cam4.70853

**Published:** 2025-04-18

**Authors:** Joviana Farhat, Lara Alzyoud, Mohammad AlWahsh, Animesh Acharjee, Basem Al‐Omari

**Affiliations:** ^1^ Department of Epidemiology and Population Health, College of Medicine and Health Sciences Khalifa University Abu Dhabi UAE; ^2^ College of Pharmacy Al Ain University Abu Dhabi UAE; ^3^ Health and Biomedical Research Center Al Ain University Abu Dhabi UAE; ^4^ Leibniz‐Institut Für Analytische Wissenschaften‐ISAS e.V. Dortmund Germany; ^5^ Institute of Pathology and Medical Research Center (ZMF) University Medical Center Mannheim Heid Elberg University Mannheim Germany; ^6^ Department of Pharmacy, Faculty of Pharmacy AlZaytoonah University of Jordan Amman Jordan; ^7^ Institute of Cancer and Genomic Sciences University of Birmingham Birmingham UK

**Keywords:** genetic testing, precision medicine, rare cancers, sequencing

## Abstract

**Background:**

Genetic testing and sequencing technologies offer a comprehensive understanding of cancer genetics, providing rapid and cost‐effective solutions. In particular, these advanced technologies play an important role in assessing the complexities of the rare cancer types affecting several systems including the bone, endocrine, digestive, vascular, and soft tissue. This review will explore how genetic testing and sequencing technologies have contributed to the identification of biomarkers across several rare cancer types in diagnostic, therapeutic, and prognostic stages, thereby advancing PM.

**Methods:**

A comprehensive literature search was conducted across PubMed (MEDLINE), EMBASE, and Web of Science using keywords related to sequencing technologies, genetic testing, and cancer. There were no restrictions on language, methodology, age, or publication date. Both primary and secondary research involving humans or animals were considered.

**Results:**

In practice, fluorescence in situ hybridization, karyotype, microarrays and other genetic tests are mainly applied to identify specific genetic alterations and mutations associated with cancer progression. Sequencing technologies, such as next generation sequencing, polymerase chain reaction, whole genome or exome sequencing, enable the rapid analysis of millions of DNA fragments. These techniques assess genome structure, genetic changes, gene expression profiles, and epigenetic variations. Consequently, they help detect main intrinsic markers that are crucial for personalizing diagnosis, treatment options, and prognostic assessments, leading to better patient prognosis. This highlights why these methods are now considered as primary tools in rare cancer research. However, these methods still face multiple limitations, including false positive results, limited precision, and high costs.

**Conclusion:**

Genetic testing and sequencing technologies have significantly advanced the field of rare cancer research by enabling the identification of key biomarkers for precision diagnosis, treatment, and prognosis. Despite existing limitations, their integration into clinical and research fields continues to improve the development of personalized medicine strategies for rare and complex cancer types.

## 1 Background

Rare cancers make up nearly a quarter (22%) of all cancer diagnosis and deaths worldwide, with a yearly incidence of less than 6 per 100,000 individuals [1]. Rare cancers comprise a highly diverse group of diseases [2]. This diversity is further stratified based on the distinct histological and molecular subtypes within these groups [2]. For example, rare bone tumors (RBTs) account for 5%–10% of rare cancer cases and are known for their unique diagnostic signatures [3]. Current research aims at detecting biomarkers and subtypes of RBT in order to explore effective treatment options [3]. Rare endocrine tumors also arise from various body organs and pose unique challenges in diagnosis and management due to their heterogeneity [4, 5]. Soft tissue tumors are a rare type of cancer, representing less than 1% of all malignancies [6]. These tumors are often difficult to evaluate and treat due to their varied body locations, characteristics, and subtypes [7, 8]. Moreover, vascular tumors are a rare subset of digestive system cancers, accounting for 0.12% to 0.28% of all digestive diseases, often leading to misdiagnosis and failure in treatment plans [9]. Other uncommon cancer types include olfactory neuroblastoma and thymic carcinoma [10, 11], which are still under investigation to establish optimal diagnostic and therapeutic strategies. The rare, aggressive, and drug‐resistant nature of these tumors makes it difficult for healthcare professionals (HCPs) to reach a therapeutic decision, reflecting the lack of diagnosis and treatment guidelines for all rare cancers [12].

As cancer is considered “a disease of the genome”, detecting a potential genetic predisposition or verifying the presence of a pathogenic gene mutation presents a variety of challenging issues for patients, their families, and HCPs [13]. In modern practice, precision medicine (PM) and genetic testing has become increasingly prevalent, offering new avenues for addressing these challenges [14]. The application of PM could help in prescribing medications that are expected to benefit a subgroup of patients whose cancer presents particular molecular or cellular features, genetic mutations, and changes in gene or protein expression patterns [15]. Multiple genetic testing technologies are also used for mapping specific genes related to the progression of cancer. For example, traditional cytogenetics assays such as fluorescence in situ hybridization (FISH) and Karyotyping, are the most often used technologies for detecting chromosomal alteration [16, 17]. In some settings, DNA Microarray have been employed as either chromosomal microarrays to identify copy number variants (CNVs) or as genotyping arrays to examine single‐nucleotide polymorphisms (SNPs) [18].

In recent years, the growing clinical demand for personalized treatment options has driven the adoption of advanced approaches, including sequencing technologies [19]. Next‐generation sequencing (NGS), also known as parallel sequencing, is currently the standard of care for patients with advanced solid tumors [20]. Consequently, a single NGS test is able to sequence all mutational types in hundreds to thousands of genes at a relatively short period and low cost [21, 22]. This emphasizes the high potential of NGS to provide the most thorough genetic investigation of cancers [20]. Through the use of NGS, both RNA and DNA sequences may be acquired [23]. DNA sequencing is classified into three types: whole‐genome sequencing (WGS), whole‐exome sequencing (WES), and targeted sequencing depending on the choice of the targeted genes for a particular condition. In parallel, RNA sequencing can identify alternative gene‐spliced transcripts, short and long non‐coding RNA sequences, gene fusion, posttranscriptional modifications, mutations/SNPs, and changes in gene expression [24, 25].

The use of genetic testing and sequencing technologies (GTST) in cancer treatment has shifted clinicians' perspective from therapeutic decisions depending on tumor location and histology toward ones focusing on molecular profiling data as well as tumor histology and location [26]. Therefore, applying sequencing technologies in conjunction with conventional clinical and pathological tests can achieve highly effective means to improve diagnosis, clinical outcomes' prediction, and prognosis [27]. This can allow HCPs to better understand the application of GTST, especially during the early detection phase [28]. Additionally, evaluating the genetic dysregulation of intrinsic pathways associated with tumorigenesis can aid in uncovering several potential therapeutic targets that may be used for personalizing the treatment of rare cancers [27]. These genomic assessments can also lead to the discovery of features that can be rapidly translated into diagnostics and monitoring strategies [13, 28, 29].

**FIGURE 1:**
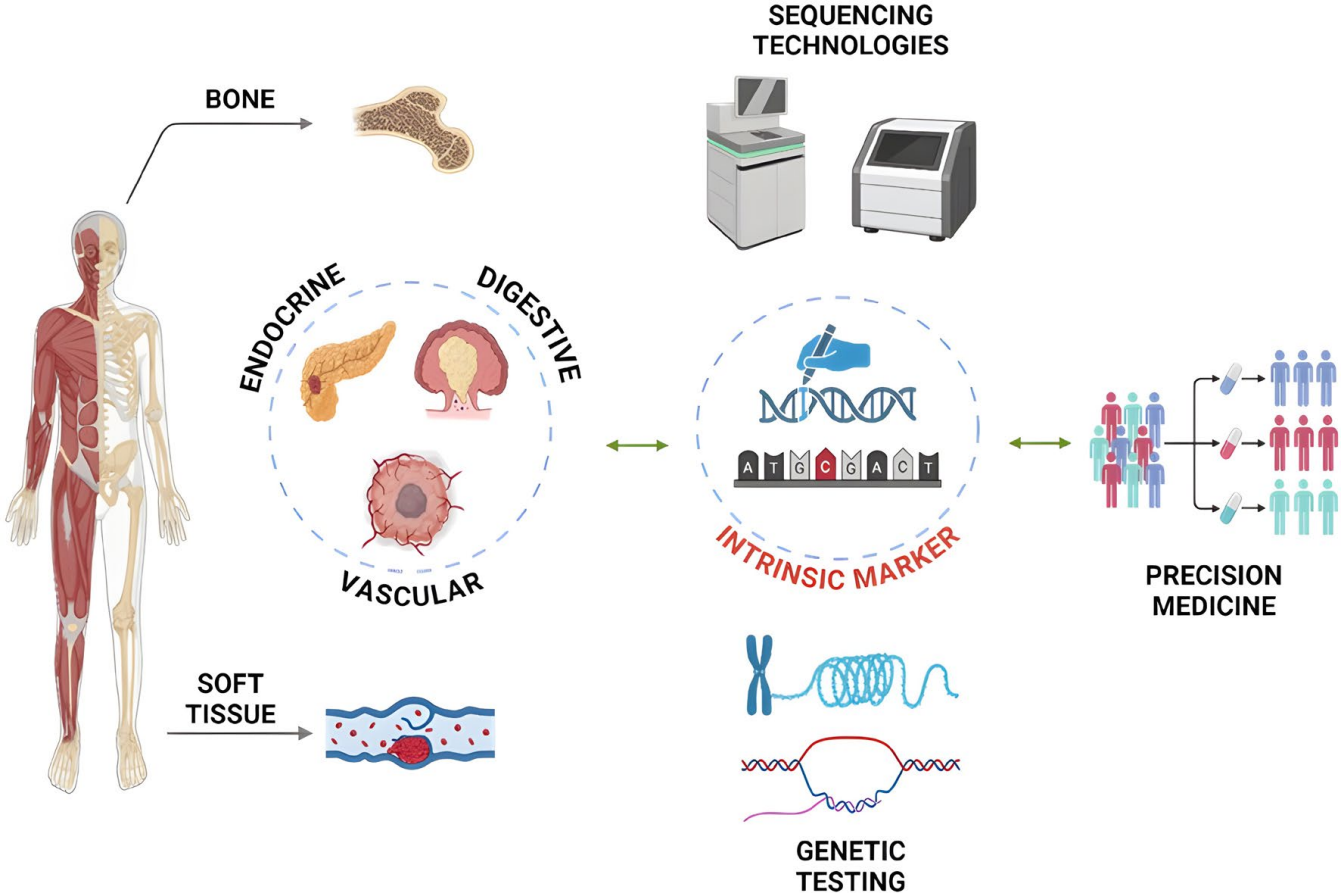
The role of GTST in rare cancers: Identifying markers and advancing PM.

Therefore, this review will explore how GTST have contributed to the identification of intrinsic markers across diverse rare cancer types in diagnostic, therapeutic, and prognostic stages, thereby advancing PM (see Figure 1).

## 2 Materials and Methods

Literature searches utilizing PubMed (MEDLINE), EMBASE, and Web of Science were conducted using terms related to sequencing, genetic testing, and cancer were used. The following key terms were used, (sequencing OR “sequencing technology” OR “advanced sequencing” OR “new sequencing” OR “novel sequencing”) AND (“genetic test” OR “genetic testing” OR “gene testing” OR gene OR “gene sequence” OR “genetic sequencing” OR “advanced genes” OR “new genes” OR “active genes” OR “novel genes”) AND (cancer OR “rare cancer” OR “rare tumors” OR “bone cancer” OR “bone tumor” OR “vascular tumor” OR “vascular cancer” OR “endocrine tumor” OR “endocrine cancer” OR “digestive cancer” OR “digestive tumor” OR “soft tissue tumor” OR “soft tissue cancer”). No language, research methods, age or date restrictions were applied. Primary and secondary research of human or animal were considered.

LibreOffice software was used to develop the tables in this manuscript to support high‐quality data presentation and differentiation. Figures were designed and structured using Biorender software and PowerPoint.

## 3 The Role of GTST in Precision Oncology: Types and Properties

In current practice, GTST have been considered a key component in advancing genomics research and offering flexible capabilities for analyzing intrinsic markers [30].

The application of NGS enabled researchers to evaluate DNA and RNA molecules in a high‐throughput manner [31, 32], with a study demonstrating the ability to process up to 500 cases per year in a cost‐effective manner [33]. Other sequencing technologies including polymerase chain reaction (PCR) and Sanger sequencing are used for amplifying DNA into millions of copies [34, 35]. WGS is applied for detecting nearly all nucleotide sequences of an individual's DNA [36]. In some cases, it requires a longer time, higher cost, and may detect non‐coding sections of DNA known as introns [36]. This expanded the use of WES that can sequence exomes (coding regions) at a much greater depth for a lower cost [37]. The analysis of genome‐wide DNA methylation at a single‐base resolution can also be achieved through Whole‐genome bisulfite sequencing (WGBS) with high accuracy and cost‐effectiveness [38]. Moreover, Chromatin Immunoprecipitation sequencing (ChIP‐seq) constitutes an advanced approach for detecting DNA binding sites for particular proteins on a genome and assessing protein‐to‐DNA interactions [39]. When paired with NGS, ChIP‐seq allows a powerful identification of genome‐wide DNA binding sites for transcription factors and other proteins [40].

It is worth mentioning that the implementation of genetic testing in different resolutions can further allow the investigation of different types of genetic variation [41]. The FISH visualizes specific small or large chromosomal regions [42]. However, obtained results are usually subject to further confirmation by either karyotype or microarray to prevent genetic mosaicism [42]. Karyotype is considered the gold standard test that can assess the entire set of chromosomes in terms of number and structure and measure “mesenchymal cells” resembling “fetal cells” while small abnormalities are sometimes not detected [43]. This emphasizes the value of Microarrays in evaluating chromosomal abnormalities with low resolution (chromosomal type) and nucleic acid sequence in terms of nucleotide order (genotypic type) [44]. Microarrays allow high output identification of multiple variant types at one time, but their high cost can be challenging [45]. Table 1 summarizes the types of GTST currently used in practice, along with their unique functions and differential characteristics.

**TABLE 1:** Overview of sequencing technologies and genetic tests.

	Function	Properties	Advantages	Disadvantages	References
Sequencing technology					
Next‐generation sequencing (NGS)	Parallel sequencing of millions of DNA fragments	Short‐read sequencing	Low cost; high precision; most widely employed approach	Limited read lengths; difficulties in repetitive regions	[213]
Long‐read sequencing	Sequencing of longer DNA fragments	Real‐time sequencing	High accuracy for structural variants; suitable for large repetitive regions	High cost, low precision; unsuitable for many applications	[214]
Polymerase chain reaction (PCR)	Amplification of DNA sequences into millions of copies	Exponential addition of nucleotide	Rapid amplification of targeted regions; cost‐effective	Risk of contamination; need for a prior knowledge of sequence	[215]
Sanger sequencing	DNA sequencing of specific regions	Chain termination method	Gold standard for small regions; high accuracy for specific applications (used to rule out a false positive result)	Time‐consuming for large‐scale sequencing; higher cost per base compared to NGS	[41]
Whole‐genome sequencing (WGS)	Detection of nearly all nucleotides sequence in an individual's DNA	Complete genome coverage	Comprehensive data; ideal for rare variant and introns (non‐coding sections of DNA) detection	Time‐consuming; high cost; challenging data storage and interpretation	[214]
Whole‐exome sequencing (WES)	Sequencing of exomes (formed by exons or coding regions)	Targeted sequencing	Sequencing at a greater depth for a lower cost; suitable for coding variant detection	Non‐coding regions are missed; limited interpretation of protein‐coding regions	[214]
Whole‐genome bisulfite sequencing (WGBS)	Analysis of genome‐wide DNA methylation at a single‐base resolution	Epigenetic analysis	High accuracy for methylation patterns; cost‐effective for DNA methylation trials	Intensive library preparation; high computational demand	[41]
Chromatin Immuniprecipitation sequencing (ChIP‐seq)	Detection of DNA‐protein binding sites and interactions	Protein‐DNA interaction mapping	Powerful identification of transcription factor binding sites; genome‐wide applicability when paired with NGS	High‐quality antibodies required; challenging library complexity	[41]
Genetic test					
FISH	Visualization of a specific small or large chromosomal regions	Cytogenetic visualization	Rapid detection of specific rearrangements	Inconclusive results (confirmation needed by karyotype or microarray for a definitive diagnosis); subject to genetic mosaicism	[216]
Karyotype	Examination of the number and structure of the entire set of chromosomes	Gold standard test	Gold standard for structural abnormalities; measurement of “mesenchymal cells” resembling “fetal cells”	Limited detection of small abnormalities	[217]
Microarrays	High‐resolution analysis of chromosomal and genomic variants	Genome‐wide assessment	High throughput; simultaneous identification of multiple variant types	High cost; limited sensitivity to structural variants	[217]
DNA methylation array	Profiling DNA methylation patterns	Epigenetic profiling	Identifies epigenetic markers; useful for cancer detection	Limited to preselected methylation sites	[218]
CRISPR diagnostics	Targeted detection of DNA or RNA sequences	CRISPR‐Cas based system	High specificity and sensitivity; rapid detection; adaptable for multiple applications	Relatively new technology; requires further validation for widespread use	[219]
Single‐cell RNA sequencing (scRNA‐seq)	Profiling gene expression at the single‐cell level	Single‐cell resolution	High sensitivity to cell‐specific expression; identifies rare cell populations	High cost; complex library preparation; challenging computational requirements	[204]

## 4 Application of GTST in Rare Cancer Tumors

### 4.1 Rare Bone Tumors

#### 4.1.1 Adamantinoma

Adamantinoma is a low‐grade, invasive bone tumor that affects the tibia or fibula and, in rare cases, other long bones [46]. Histopathological examination is still the primary method for diagnosing adamantinoma. Several studies suggested that this condition is of epithelial origin due to a possible connection between adamantinoma and tumors emerging from the epithelium, such as osteofibrous dysplasia or synovial sarcoma [47]. Adamantinoma and osteofibrous dysplasia‐like (ODF‐like) adamantinoma both possess comparable histological features and are not easily distinguishable when making a diagnosis. Previous cytogenetic analysis of both conditions identified a recurring pattern of numerical anomalies, including additional copies of chromosomes 7, 8, 12, 19, and 21 in both variants [48, 49]. The application of WES and RNA sequencing (RNA‐Seq) on both tumor types resulted in the recurrent mutation of Lysine Methyltransferase 2D (KMT2D) gene in 38% of adamantinomas, indicating the potential role of chromatin structure and integrity in adamantinoma carcinogenesis. RNA‐Seq analysis also revealed a novel somatic gene fusion in an adamantinoma, which could be used as a diagnostic marker [50]. Although adamantinoma and synovial sarcoma of the tibia share morphologic and immunophenotypic similarities, SS18 translocation is a known marker of Synovial sarcoma. Therefore, conducting FISH to investigate the status of SS18 translocation can exclude synovial sarcoma and is recommended before diagnosing spindle cell adamantinoma [51]. Recently, a study revealed that DNA methylation could differentiate between Adamantinoma‐Like Ewing sarcoma and the conventional Ewing sarcoma based on the distinct methylation signature between both tumors [52, 53, 54].

Despite ongoing findings in adamantinoma, the potential to generalize findings is still limited due to small sample sizes and a lack of validation in diverse populations. False positive results may still occur in FISH testing, requiring the use of more robust and standardized diagnostic guidelines. Integrating tools such as KMT2D and methylation profiling could improve diagnostic precision. Moreover, validating these findings in larger studies could facilitate their use in targeted therapies or clinical trials.

#### 4.1.2 Chondromyxoid Fibroma

Chondromyxoid fibromas (CMF) arise from cartilaginous joints and are associated with a low risk of metastasis [55]. Due to the limited number of documented cases, limited data is known about the etiology and pathogenesis of these benign tumors [56, 57]. Although surgery is widely considered to be the mainstay of treatment, adjuvant radiation therapy, intra‐lesional curettage, and cementation represent efficient therapeutic alternatives that provide excellent outcomes with a low recurrence rate [55, 58]. Further insights were provided through the successful implementation of whole‐genome mate‐pair sequencing and RNA‐Seq technologies in CMF, which identified the overexpression of the glutamate receptor gene (GRM1) in several rare cases that lead to disordered glutamate signaling [59]. These findings provide evidence for the potential of the GRM1 gene to be a promising therapeutic target for the treatment of CMF [60]. However, the limited case numbers and reliance on individual studies restrict the generalizability of thefindings.

Futuristically, multicenter studies and preclinical models are essential to confirm GRM1's therapeutic potential. This highlights the need to incorporate sequencing technologies in regular diagnostics to identify targets and improve treatment strategies for this rare tumor.

#### 4.1.3 Chordoma

Chordomas are tumors that originate from the notochord at the base of the skull and spinal cord, and are often adequately treated with surgery and radiotherapy [61, 62]. Due to their high metastatic potential and recurrence rates, genetic abnormalities are suggested to be the cause behind the formation of these tumors [63]. While there is no conclusive genetic marker established for chordomas, classical G‐banding, genome‐wide oligonucleotide microarrays, comparative genomic hybridization (CGH), and FISH have detected several genes implicated in tumor formation [64, 65]. Genetic aberrations in the brachyury gene (TBXT) have been noticed through Sanger sequencing and WES [66]. Other specific mutations obtained through whole‐exome and whole‐genome NGS have resulted in a treatment strategy that is centered on targeting these anomalous genes [67, 68, 69].

The variation in genetic findings along with the absence of a definitive biomarker highlights the need for more directed studies about chordomas. This can be explained due to the high costs and limited accessibility of genome‐wide analyses in practice. Therefore, a comprehensive genomic analysis of a patient's tumor can allow the development of a personalized treatment plan.

#### 4.1.4 Periosteal Osteosarcoma

Periosteal osteosarcoma (PO) is a rare subtype of bone tumors [70]. Compared to osteosarcoma, periosteal osteosarcoma has a minimal risk of metastasis and a better overall prognosis [71]. Only high‐grade tumors might require adjuvant treatment with chemotherapy, while low‐grade tumors are managed with wide excision [72]. In two patients, a missense mutation resulting in the inactivation of at least one allele in the Tumor Protein 53 (TP53) gene was revealed through WES. As TP53 is one of the most commonly mutated genes in human cancers, this mutation is believed to be directly associated with the pathogenesis of periosteal osteosarcoma [73, 74]. These findings align with earlier research, where point mutations in TP53 have been detected through polymerase chain reaction‐Single‐Strand Conformation Polymorphism (PCR‐SSCP) of exons 4–8 followed by Direct Genomic Sequencing, revealing their role in the early progression of malignant osteoblastic tumors. In some cases, complex chromosomal aberrations were observed, but these have not been particular to periosteal osteosarcoma but rather to all types of sarcomas [70].

Therefore, limited evidence requires larger studies to obtain a clear genetic basis. The lack of subtype‐specific markers also limits the development of targeted therapies. Consequently, exploring TP53‐specific treatments in clinical studies and conducting longitudinal clinical trials is necessary to identify biomarkers for more effective and personalized PO management strategies.

### 4.2 Rare Endocrine Tumors

#### 4.2.1 Adrenocortical Carcinoma

Adrenocortical Carcinoma (ACC) is a rare endocrine malignancy characterized by a poor prognosis [75]. Most ACC cases are characterized by steroid hormone excess or abdominal mass; nevertheless, 15% of these cases are discovered by chance. Advances in genomics have enabled molecular characterization of ACC tumors, revealing multiple complex and inconsistent genetic alterations [76, 77]. This has been of great interest due to the poor prognosis and high mortality rates of ACC secondary to metastasis [78]. Consequently, sequencing technologies have enabled the identification of novel targets for metastatic ACC, which may improve patient outcomes and prognosis based on findings from nine patient samples [79]. NGS identified mutations in the TP53, Neurofibromin 1 (NF1), Cyclin‐Dependent Kinase Inhibitor 2A (CDKN2A), Multiple Endocrine Neoplasia type 1 (MEN1), Catenin Beta 1 (CTNNB1), and Ataxia‐Telangiectasia Mutated (ATM) genes to be the most common [80, 81, 82]. Interestingly, another study identified Erb‐B2 Receptor Tyrosine Kinase 4 (ERBB4) as a frequently mutated gene in metastatic ACC through a combination of WES and Sanger sequencing [83]. Another analysis evaluated tumors using various WGS, WES, DNA microarrays, PCR, and Sanger sequencing [78, 84, 85]. These studies identified TP53 and CTNNB1 mutations in a significant percentage of samples. Other driver genes were also identified through WES and DNA microarrays [78, 84, 85].

The validation of these findings through large‐scale studies, along with an assessment of their utility across various populations, is essential. Clinically, integrating these mutations in routine practice could enable customized treatment strategies, especially for metastatic cases. In the long term, this approach may improve patient outcomes and prognosis through earlier and more precise interventions.

#### 4.2.2 Pheochromocytoma and Paraganglioma

Pheochromocytomas and paragangliomas (PPGLs) are neuroendocrine tumors arising from sympathetic or parasympathetic tissues [86]. Because this rare type of tumor can be lethal, early detection is crucial [87]. In some cases, the non‐specific symptoms of PPGLs can be associated with diagnostic and therapeutic delays [88, 89]. Approximately 40% of these sporadic tumors originate from inherited genetic mutations, making them some of the most strongly hereditary human tumors [90]. Different sequencing technologies can be used to detect these mutations; yet, NGS along with Sanger sequencing are mainly applied [91, 92, 93]. Typical genetic screening for PPGLs involves the identification of the most common pathogenic mutation [94]. Consequently, it is recommended to make genetic testing more accessible to PPGLs patients and their families for early detection and intervention of this aggressive cancer [86].

In clinical practice, it can be suggested that intrinsic biomarkers detected through genetic testing can help HCPs to better evaluate disease progression by classifying these markers into diagnostic, therapeutic, and prognostic categories for better disease management and patient prognosis (see Table 2). However, limited access to sequencing technologies and reliance on common mutations restrict the detection of novel biomarkers.

**TABLE 2:** Diagnostic, therapeutic, and prognostic markers in rare bone and endocrine tumors.

Type of cancer	Sequencing technology	Gene translocation or fusion	Frequently mutated genes	Type of biomarkers	References
Rare bone tumors					
Adamantinoma	WES and RNA‐Seq	EPHB4‐MARCH10 gene fusion	KMT2D	Diagnostic markers	[50]
Chondromyxoid fibroma	Whole‐genome mate‐pair sequencing and RNA‐seq	—	GRM1	Therapeutic target	[60]
Chordoma	WES, WGS and Sanger sequencing	—	TBXT, CDK4, PBRM1, ERBB3, FGFR1, ATM, CDKN2A and CHEK2	Therapeutic target	[66, 67, 68]
Periosteal osteosarcoma	WES, PCR, and Direct Genomic Sequencing	—	TP53	Prognostic marker	[70, 73, 220, 221]
Rare endocrine tumors					
Adrenocortical Carcinoma	WES, WGS, Sanger sequencing, DNA microarrays, PCR and NGS	—	TP53, NF1, CDKN2A, MEN1, CTNNB1, ATM, ERBB4, PRKAR1A, RPL22, TERF2, ZNRF3 and CCNE1	Therapeutic target	[78, 80, 83, 84, 85, 222, 223]
Paraganglioma and pheochromocytoma	NGS and Sanger Sequencing	—	SDHA, SDHB, SDHC, SDHD, SDHAF2, FH, VHL, RET, NF1, MAX, TMEM127, and KIF1B	Diagnostic marker	[91, 92, 94, 224]

Abbreviations: ATM, Ataxia Telangiectasia Mutated; CCNE1, Cyclin E1; CDK4, Cyclin‐Dependent Kinase 4; CHEK2, Checkpoint Kinase 2; EPHB4‐MARCH10, Ephrin type B receptor 4‐ Membrane‐Associated RING‐CH 10; ERBB3, Erb‐B2 Receptor Tyrosine Kinase 3; FGFR1, Fibroblast Growth Factor Receptor 1; FH, Fumarate Hydratase; KIF1B, Kinesin Family Member 1B; PBRM1, Polybromo 1; PRKAR1A, Protein Kinase, cAMP‐Dependent, Regulatory, Type I, Alpha; RET, Rearranged during Transfection; RPL22, Ribosomal Protein L22; SDHA, Succinate Dehydrogenase Complex Flavoprotein Subunit A; SDHAF2, Succinate Dehydrogenase Complex Assembly Factor 2; TERF2, Telomeric Repeat Binding Factor 2; TMEM127, Transmembrane Protein 127; VHL, Von Hippel–Lindau; ZNRF3, Zinc and Ring Finger 3.

### 4.3 Rare Digestive System Tumors

#### 4.3.1 Appendiceal Cancer

**FIGURE 2:**
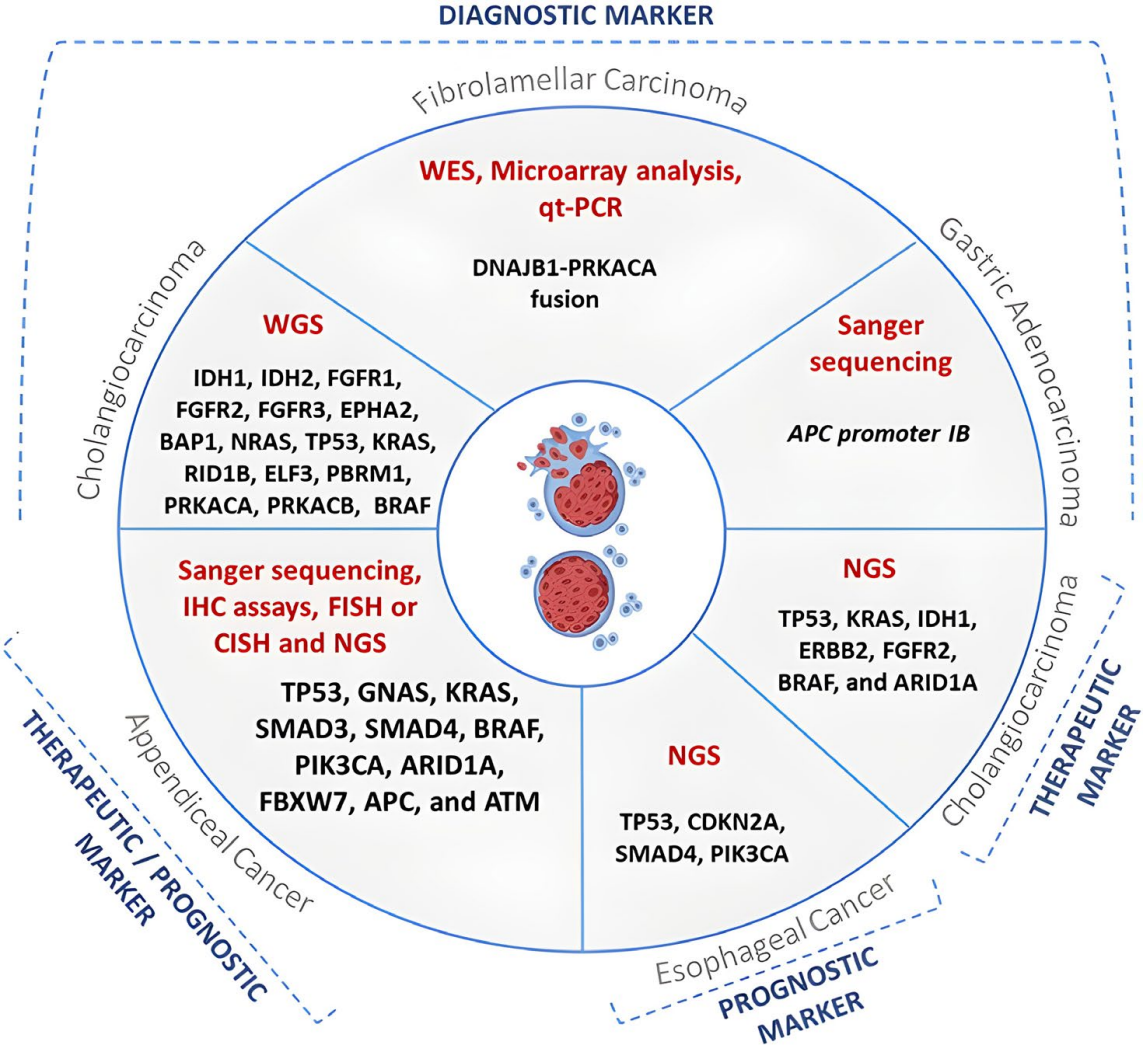
Classification of biomarkers in rare digestive system tumors based on GTST. APC, Adenomatous Polyposis Coli; ARID1A, AT‐Rich Interaction Domain 1A; ATM, Ataxia‐Telangiectasia Mutated; BAP1, BRCA1‐Associated Protein 1; BRAF, v‐Raf Murine Sarcoma Viral Oncogene Homolog B; CDKN2A, Cyclin Dependent Kinase Inhibitor 2A; CISH, Chromogenic In Situ Hybridization; DNAJB1, DnaJ Heat Shock Protein Family (Hsp40) Member B1; ELF3, E74‐like factor 3; EPHA2, Ephrin Type‐A Receptor 2; ERBB2, Erythroblastic Oncogene B2; FBXW7, F‐box and WD repeat domain containing 7; FGFR1, Fibroblast Growth Factor Receptor 1; FISH, fluorescence in situ hybridization; GNAS, Guanine Nucleotide‐Binding Protein G(s) Alpha Subunit; IDH1, Isocitrate Dehydrogenase 1; IDH1, Isocitrate Dehydrogenase 1; IHC, immunohistochemistry; KRAS, Kirsten Rat Sarcoma; NGS, next‐generation sequencing; NRAS, Neuroblastoma RAS; PBRM1, Polybromo 1; PIK3CA, phosphatidylinositol‐4,5‐bisphosphate 3‐kinase catalytic subunit alpha; PRKACA, Protein Kinase, cAMP‐Dependent, Catalytic, Alpha; qt‐PCR, quantitative real‐time polymerase chain reaction; RID1B, RAD54‐like protein 1B; SMAD4, SMAD family member 4; TP53, Tumor Protein 53; WES, whole‐exome sequencing; WGS, whole‐genome sequencing.

Appendiceal cancer is a rare malignancy that is often discovered incidentally following appendectomy for acute appendicitis [95]. For this type of cancer, surgical therapy is the cornerstone of treatment. In advanced tumor cases or when ineligible for surgical resection, treatment primarily includes chemotherapy and palliative care [96, 97]. NGS of all five appendiceal cancer subtypes revealed distinct somatic mutations, which are listed in Figure 2 [98, 99, 100]. The most prevalent are Rat Sarcoma (RAS), Guanine Nucleotide‐Binding Protein G(s) Alpha Subunit (GNAS), and TP53 mutations [101]. While Sanger sequencing, protein expression/immunohistochemistry (IHC), and gene amplification (FISH or Chromogenic In Situ Hybridization [CISH]) can all be used, NGS remains the preferred method for profiling appendiceal tumors [102]. Tissue‐NGS and blood‐NGS are employed interchangeably in profiling this type of cancer, as they typically yield comparable molecular profiles [100]. Note that some recommendations have been proposed to guide the use of NGS in appendiceal cancer depending on the nature of the tumor [103]. For instance, it is recommended to use blood‐NGS for low‐grade tumors and tissue‐NGS for high‐grade tumors to enhance the accuracy of predicting prognostic biomarkers and novel therapeutic targets [104].

Overall, these recommendations need further validation to identify potential therapeutic targets, and evaluate their clinical relevance in improving patient outcomes. Once validated, theses markers could be integrated into clinical protocols to develop precise prognostic and therapeutic tools tailored to tumor grade.

#### 4.3.2 Cholangiocarcinoma

Cholangiocarcinoma is a type of primary hepatic cancer that arises from the epithelium of the bile duct [105]. This tumor is divided into three subtypes: intrahepatic cholangiocarcinoma (iCCA), perihilar cholangiocarcinoma (pCCA), and distal cholangiocarcinoma (dCCA) [105]. In recent years, the increased incidence of cholangiocarcinoma has contributed to further advancements in assessing its etiology, prognosis, and clinical management. The standard of treatment is surgical excision; however, chemotherapy and external‐beam radiation therapy are the primary treatment options in advanced stages [106, 107]. The variable genomic, epigenetic, and molecular nature of cholangiocarcinoma detected by NGS has posed a greater interest in creating novel diagnostic strategies and therapies that may significantly improve patient outcomes [108, 109]. NGS and WES are also often used to identify somatic mutations in patients with iCCA, pCCA, and dCCA. Interestingly, WES identified SAV1 as a potential driver of Intrahepatic cholangiocarcinoma (ICC) [110]. For example, mutations in TP53, Kirsten Rat Sarcoma (KRAS), and AT‐Rich Interaction Domain 1A (ARID1A) genes have been associated with cholangiocarcinoma [111]. Consequently, some clinical trials have been testing the effectiveness of targeted drugs specific to these genes in conjunction with chemotherapy [112]. It is worth mentioning that WGS is applied as an additional tool for differentiating cholangiocarcinoma tumors from other types of liver cancers since TP53 mutations have also been largely expressed in hepatic tumors [113]. Other mutations including Isocitrate Dehydrogenase 1 (IDH1), IDH2, Fibroblast Growth Factor Receptor 1 (FGFR1), FGFR2, FGFR3, Ephrin Type‐A Receptor 2 (EPHA2), BRCA1‐Associated Protein 1 (BAP1), and Neuroblastoma RAS (NRAS) were found in iCCA cases. In parallel, pCCA/dCCA cases were found to have TP53, KRAS, AT‐Rich Interaction Domain 1B (ARID1B), E74‐like factor 3 (ELF3), Polybromo 1 (PBRM1), Protein Kinase, cAMP‐Dependent, Catalytic, Alpha (PRKACA), PRKACB, and v‐Raf Murine Sarcoma Viral Oncogene Homolog B (BRAF) mutations.

This reveals the heterogeneous nature of cholangiocarcinoma, emphasizing the need for subtype‐specific diagnostic and therapeutic strategies [114]. Expanding genomic studies to include diverse populations and integrating molecular profiling into routine care is also required, especially to improve survival rates.

#### 4.3.3 Esophageal Cancer

Esophageal cancer is known to be a fatal disease with a low survival rate despite medical treatment [115]. Most malignant esophageal neoplasms are classified as adenocarcinoma or squamous cell carcinoma based on tumor symptoms, histology, and morphology [116]. Depending on the tumor type, treatment may include esophagectomy, endoscopic mucosal excision, chemotherapy, or chemoradiotherapy [115]. The high mortality rates reported have fueled extensive efforts to obtain a genome‐wide molecular profile of esophageal tumors [117], with NGS being widely implemented [118]. Accordingly, the most prevalent pathogenic mutations were linked to TP53, CDKN2A, SMAD family member 4 (SMAD4), and Phosphatidylinositol‐4,5‐bisphosphate 3‐kinase catalytic subunit alpha (PIK3CA) genes [119]. In the long term, SMAD4 and TP53 mutations will be classified as predictive biomarkers for tumor recurrence and poor prognosis [120]. A recent study also discussed the potential of long‐read and single‐cell DNA sequencing technologies to better predict clonal evolution in esophageal adenocarcinoma [121].

Consequently, additional efforts should focus on standardizing genomic profiling techniques and incorporating them into treatment protocols to facilitate earlier intervention and improved disease management, especially given the high mortality rate associated with esophageal cancer.

#### 4.3.4 Fibrolamellar Carcinoma

Fibrolamellar hepatocellular carcinoma (FLHCC) is a rare type of liver cancer that primarily affects young adults and teenagers without underlying liver disorders [116]. FLHCC symptoms often include non‐specific stomach pain, malaise, nausea, and weight loss. Due to the chemotherapy‐resistant nature of FLC, surgical procedures are the only effective and main cornerstone of FLC management [122]. Consequently, the genomic profiling of FLHCC tumors has consistently revealed the presence of a fusion between DnaJ Heat Shock Protein Family (Hsp40) Member B1 (DNAJB1) and Protein Kinase, cAMP‐Dependent, Catalytic, Alpha (PRKACA) [123]. The detection of the DNAJB1‐PRKACA fusion constitutes a unique marker in FLCC tumors specifically, as evidenced by WES, microarray analysis, and quantitative real‐time polymerase chain reaction (qt‐PCR) [124, 125]. Moreover, WGS of the FLHCC specimen has identified a few genomic alterations located on the lower end of the mutational spectrum. As the genomic landscape of FLHCC lacks a second‐hit mutation, the DNAJB1‐PRKACA fusion protein is the optimal target for diagnostic and therapeutic improvements [126, 127].

The detection of the DNAJB1‐PRKACA fusion as a basis for FLHCC constitutes a key component for understanding this rare cancer. However, the lack of other genomic alterations in FLHCC limits the scope for developing a targeted therapy. Future research should further evaluate the biological mechanisms of this fusion protein for earlier detection opportunities and personalized treatment approaches.

#### 4.3.5 Gastric Adenocarcinoma and Proximal Polyposis of the Stomach

Gastric adenocarcinoma and proximal polyposis of the stomach (GAPPS) is a rare, hereditary cancer that belongs to familial adenomatous polyposis [75]. This cancer tumor has an autosomal dominant inheritance pattern and carries a high risk of progressing into gastric adenocarcinoma [128]. It is unclear if diagnostic and therapeutic guidelines are established for this cancer. Several retrospective case reports rely on gastroscopy and biopsies as diagnostic tools and recommend prophylactic gastrectomy [128, 129, 130]. The use of Sanger sequencing revealed alterations in the Adenomatous Polyposis Coli (APC) promoter IB as an underlying genetic driver of GAPPS [131, 132].

Due to its hereditary nature, new criteria for early detection, prophylactic treatment, and the formation of prospective family registries must be implemented for future research investigations [133, 134]. Therefore, intrinsic markers are considered a key component during diagnosis, treatment, and prognosis to achieve better clinical outcomes and quality of life (see Figure 2).

### 4.4 Rare Vascular Tumors

#### 4.4.1 Angiosarcoma

Angiosarcoma is known as an aggressive endothelial tumor that can arise at any site within the body, while the cutaneous lesions localized in the head and neck account for 60% of angiosarcomas [135]. Despite the limited data available regarding the pathogenesis of angiosarcomas, some risk factors were identified, including chronic lymphoedema, radiation exposure, environmental toxins, and some familial disorders [136]. In practice, WES analysis of 47 tumors revealed that Angiosarcoma of the head, neck, face, and scalp (HNFS) is closely related to a higher tumor mutation burden (TMB) and a dominant ultraviolet damage mutational signature [137]. This implies that immune checkpoint inhibitors may be a valuable therapeutic option for a subset of patients with angiosarcoma of HNFS [138]. The application of WES and RNA‐Seq on tumor samples also revealed six missense mutations in Neuroblastoma Breakpoint Family Member 10 (NBPF10), NBPF15, Zinc Finger Protein 678 (ZNF678), Vacuolar Protein Sorting 8 (VPS8), Piccolo Presynaptic Cytomatrix Protein (PCLO), and ATP Binding Cassette Subfamily B Member 1 (ABCB1) genes. These mutations resulted in an amino acid shift toward hydrophobicity, which tends to be related to immunogenic neo‐peptides and immune checkpoint inhibition therapy [139, 140]. Angiosarcomas were found to be genetically diverse tumors with a wide variety of genetic anomalies. WGS across 18 samples from the head and neck detected mutations in TP53, Kinase Insert Domain Receptor (KDR), Protein Tyrosine Phosphatase, Receptor Type B (PTPRB), and Protection of Telomeres 1 (POT1) genes [141]. Similarly, NGS identified mutations in TP53 and POT1 genes [142]. Another study utilized a hybridization‐based targeted NGS assay to detect TP53 and PTPRB mutations, as well as CDKN2A deletions [143]. These findings show that the most frequently observed genetic aberrations across cases from the head and neck areas are primarily in the TP53 gene.

Future studies should prioritize stratifying patients by genetic profiles, such as TP53 and POT1 mutations, in order to personalize treatment plans. Furthermore, expanding the sample size of genomic studies and integrating multi‐omics approaches may further refine therapeutic strategies and improve survival outcomes [144].

#### 4.4.2 Epithelioid Hemangioendothelioma

Epithelioid hemangioendothelioma (EHE) is a low‐grade malignant vascular tumor that can arise in different anatomical sites, including the liver, bone, and lungs [145, 146]. The driver mutation for 90% of EHE is the WW Domain Containing Transcription Regulator 1—Calmodulin Binding Transcription Activator 1 (WWTR1‐CAMTA1) fusion gene. The remaining 10% are due to the Yes‐Associated Protein 1—Transcription Factor E3 (YAP1‐TFE3) fusion gene, an underrecognized variant that is not well described in the literature [147, 148]. Genetic testing using FISH and/or PCR has proven to be an excellent diagnostic method for detecting EHE fusion genes [149]. Recent attempts have been made to construct a thorough molecular profile of EHE using NGS to detect genomic variants. Subsequently, these variants can be utilized to predict aggressiveness and prognosis of this condition [150]. The majority of primary EHE cases present with a small number of mutations. Genetic mutations could be detected using NGS in nearly all EHE samples examined (see Figure 1) [151]. As a result, these findings contribute to a better understanding of the molecular profile of EHE and present novel therapeutic targets.

Prospectively, NGS‐based molecular profiling can offer a promising tool for predicting tumor aggressiveness and prognosis, along with validation studies on diverse patient cohorts. This could improve early disease detection and the use of targeted therapy, especially for the more aggressive or under‐researched variants of EHE.

#### 4.4.3 Kaposiform Hemangioendothelioma

**FIGURE 3:**
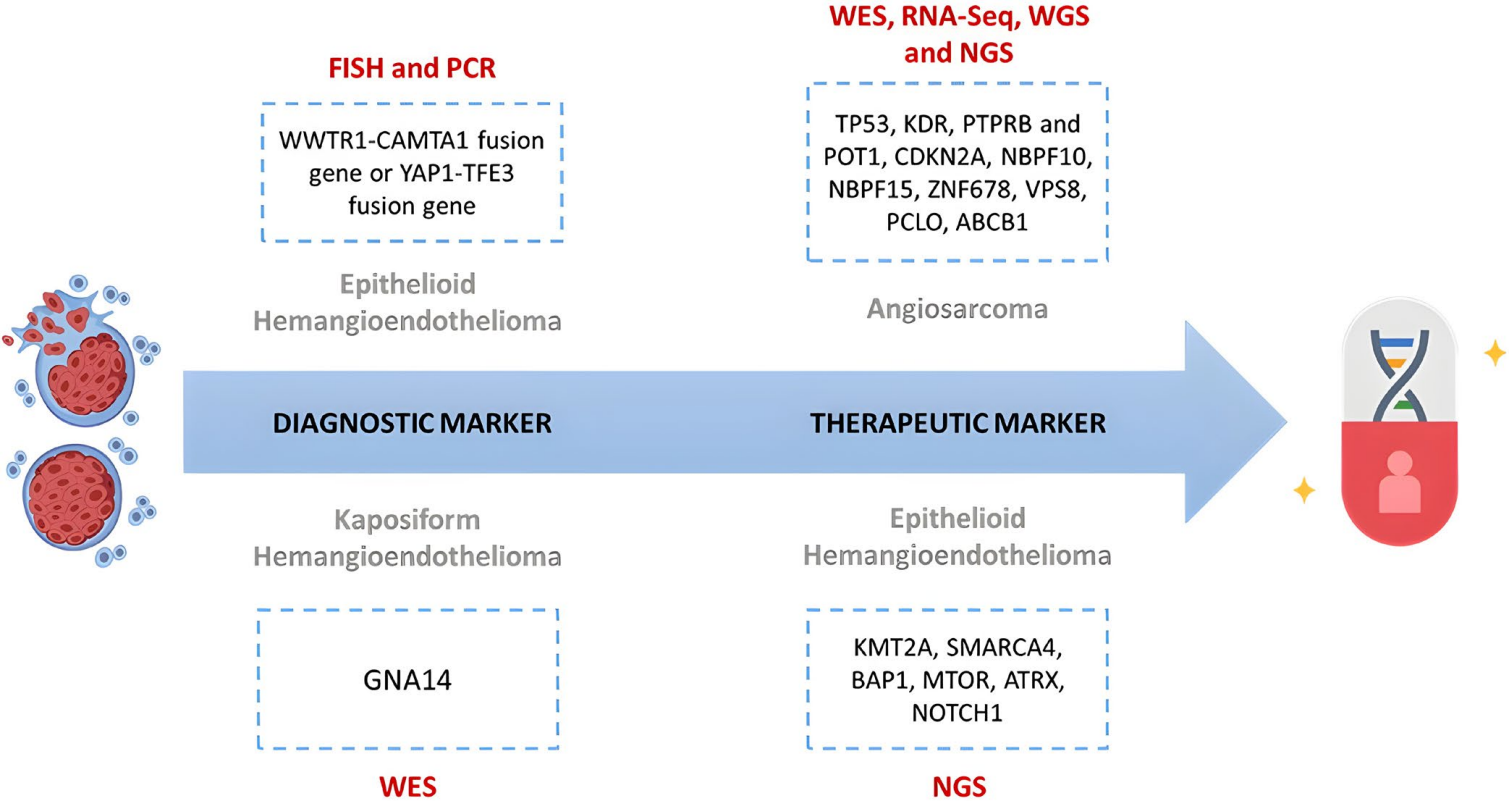
Classification of biomarkers in rare vascular tumors based on GTST. ABCB1, ATP Binding Cassette Subfamily B Member 1; ATRX, Alpha Thalassemia/Mental Retardation Syndrome X‐Linked; BAP1, BRCA1‐Associated Protein 1; CDKN2A, Cyclin Dependent Kinase Inhibitor 2A; FISH, fluorescence in situ hybridization; GNA14, G Protein Subunit Alpha 14; KDR, Insert Domain Receptor; KMT2A, Lysine Methyltransferase 2A; MTOR, Mechanistic Target of Rapamycin; NBPF10, Neuroblastoma Breakpoint Family Member 10; NGS, next‐generation sequencing; NOTCH1, Neurogenic Locus Notch Homolog 1; PCLO, Piccolo Presynaptic Cytomatrix Protein; PCR, polymerase chain reaction; PGT1, Progestogen‐Associated Endometrial Protein 1; PTPRB, Receptor Type B; SMARCA4, SWI/SNF‐related matrix‐associated actin‐dependent regulator of chromatin subfamily A member 4; TP53, Tumor Protein 53; VPS8, Vacuolar Protein Sorting 8; WES, whole‐exome sequencing; WGS, whole‐genome sequencing; WWTR1‐CAMTA1, WW Domain Containing Transcription Regulator 1—Calmodulin Binding Transcription Activator 1; YAP1‐TFE3, Yes‐Associated Protein 1—Transcription Factor E3; ZNF678, Zinc Finger Protein 678.

Kaposiform hemangioendothelioma (KHE) is a congenital, locally aggressive vascular tumor that can affect the skin, deep soft tissues, or bones, and is characterized by a poor prognosis [145, 152]. There is no treatment guideline established due to a lack of trials, and the various recommendations for patient care are based on published case series [153]. The importance of early diagnosis and treatment of KHE is related to its potential progression to the Kasabach‐Merritt phenomenon (KMP), a condition marked by life‐threatening thrombocytopenia and consumptive coagulopathy [154]. Earlier studies identified G Protein Subunit Alpha 14 (GNA14) mutations in KHE samples through WES and targeted sequencing. However, further findings showed that GNA14 mutations cannot be implicated as markers for diagnosis, as they are not entity‐specific. This is supported by a recent comprehensive mutational analysis and genome‐wide methylation profiling of KHE, which found that none of the cases had GNA14 mutations and just one case has a RAD50 Double Strand Break Repair Protein (RAD50) mutation [155]. In conclusion, rare vascular tumors are associated with various markers that can be used as diagnostic or therapeutic targets in future genomics‐based research using GTST (see Figure 3).

### 4.5 Rare Soft Tissue Tumors

#### 4.5.1 Desmoid Tumors

Desmoid tumors (DT), also known as aggressive fibromatosis, are sporadic fibroblastic proliferations [156]. The diagnosis of DTs is often incidental and challenging, especially in the absence of clinical symptoms [157]. While DTs have a relatively low mortality rate, they are severe in nature and have a high chance of recurrence [158]. Most DTs exhibit histological similarities to benign fibroblastic lesions, myofibroblastic lesions, and low‐grade sarcoma [159]. Several studies have reported the presence of somatic mutations in the Catenin Beta 1 (CTNNB1) gene in DTs and proposed CTNNB1 sequencing as a reliable diagnostic marker for the diagnosis of these tumors [160]. Direct sequencing of CTNNB1 in 260 cases of DTs and 191 cases of spindle cell lesions revealed CTNNB1 mutations in 88% of sporadic DTs, but none in all other lesions studied [159]. The use of WES and RNA sequencing identified CTNNB1 mutations in 87.5% of tumor cases. Additionally, a copy number loss in chromosome 6 (chr6) was observed in 21.9% of cases. This indicates that CTNNB1 mutations and a chr6 copy number loss are likely the causative mutations underlying the tumorigenesis of DT [161]. A similar study utilizing NGS detected the CTNNB1 serine 45 phenylalanine (S45F) mutation, which usually presents with an aggressive phenotype [157]. This is consistent with findings that identified the S45F mutation of CTNNB1 as a high‐risk factor for recurrence of DT and a predictive marker for sporadic DT [162]. Another study, applying WES, identified OTCH2 and HES1 as potential markers for evaluating the response to Imatinib [163].

Therefore, the structuration of an appropriate DT therapy is challenging, especially considering the limited number of trials and case studies available due to the rarity of the disease [164]. In most circumstances, active monitoring should be regarded as the first step in the management of DT, mainly through gene sequencing for better patient care. In the follow‐up stage, molecular profiling can aid in identifying patients who might benefit from additional therapies and carry a risk of tumor reoccurrence [165]. In the event of tumor progression, surgery and/or radiation therapy are undertaken following a thorough examination of the patient's condition [166].

#### 4.5.2 Desmoplastic Small Round Cell Tumors

Desmoplastic small round cell tumor (DSRCT) is an invasive soft tissue neoplasm composed of small round tumor cells [167]. The exact origin of this aggressive tumor is still subject to ongoing research investigations. DSRCT is rarely visualized as a single tumor; in most cases, multiple abdominal tumors are detected at the time of diagnosis. This necessitates multimodal therapy consisting of surgery, radiotherapy, and chemotherapy [168]. Therefore, genomic characterization of rare diseases such as DSRCT has been crucial for improving the assessment of defective pathways involved in tumor onset and progression [169]. A few genetic profiling studies revealed that DSRCT is associated with a specific chromosomal translocation, t(11;22)(p13;q12), involving the Ewing Sarcoma Breakpoint Region 1 (EWSR1) and Wilms Tumor 1 (WT1) genes. This translocation is detectable through WGS and aids in diagnosing DSRCT cases [170]. Another translocation of exon 9 of EWS to exon 7 of WT1 was found using reverse‐transcriptase polymerase chain reaction (RT‐PCR) analysis, emphasizing the necessity of genetic testing when diagnosing DSRCT [171]. It is worth noting that most of these studies have described individual cases, meaning that further research is needed to establish definitive diagnostic recommendations. Moreover, WES revealed that genomic dysregulations in the Mesenchymal‐Epithelial Transition/Epithelial‐Mesenchymal Transition (MErT/EMT) and DNA Damage Response (DDR) pathways, as well as the deletion of chromosome 6, play a critical role in promoting tumor recurrence [172].

Despite the recent attempts to introduce molecular targeted therapies, there are still no available interventions that target the distinctive EWSR1‐WT1 gene fusion [173]. Therefore, extending the use of molecular profiling during diagnosis and disease progression is crucial for understanding the implications of the pathogenic genetic fusion [174, 175]. Additionally, the growing data on the somatic mutations in DSCRT will open up new opportunities for future usage of appropriate targeted therapeutics [176, 177].

#### 4.5.3 Synovial Sarcoma

Synovial sarcoma (SS) is a subtype of sarcomas characterized by a translocation between SSX18 and SSX1, SSX2, or SSX4 [178, 179]. Recently, the majority of patients with SS were classified under the SS subtype I, corresponding to the high‐risk tumors [180]. This translocation is the primary initiating agent and oncogenic driver of SS tumors, which is frequently detected by FISH and PCR analysis. These disease‐specific fusion genes may also be identified by NGS‐based RNA sequencing, further demonstrating the utility and accuracy of genetic testing in the diagnosis of sarcomas [181, 182, 183]. In addition to molecular profiling, a diagnosis can be made through biopsy, Immunohistochemistry (IHC), physical examination, and Magnetic Resonance Imaging (MRI) [168]. While surgery remains the mainstay of treatment, the most recent work has focused on finding other genetic alterations in SS tumors for targeted therapies [168]. The NGS of 409 cancer‐related genes has identified eight somatic mutations in SS tumors and was later confirmed by Sanger sequencing [184].

Overall, the detected mutations present new markers for the discovery of targeted therapies in multiple types of rare soft tissue tumors [184]. Parker and colleagues supported the role of FYN proto‐oncogene, Src family tyrosine kinase (FYN) in suppressing anti‐cancer activity through SS18‐SSX functional inhibition in order to improve the effectiveness of genetic and histone deacetylase inhibitor (HDACi) treatment against synovial sarcoma [185]. In a clinical setting, the use of GTST can help HCPs to approach the main markers, either diagnostic, therapeutic, or prognostic, which are directly related to cancer progression and specific for each individual (see Table 3).

**TABLE 3:** Diagnostic, Therapeutic, and Prognostic Markers in rare soft tissue tumors.

Type of cancer	Sequencing technology	Gene translocation or fusion	Frequently mutated genes	Type of biomarkers	References
Sclerosing epithelioid fibrosarcoma	WES, NGS, SNP array analysis, RNA sequencing, IHC analysis, FISH and rt‐PCR	FUS gene fusion or EWSR1 fusion or YAP1‐KMT2A fusion		Prognostic markers	[1, 2, 3]
Desmoid tumor	Direct sequencing, WES, RNA‐Seq and NGS	—	*CTNNB1*	Diagnostic and Prognostic marker	[4, 5, 6, 7]
Schwannoma	WES and RNA‐seq	SH3PXD2A‐HTRA1 fusion	NF2	Prognostic markers	[8, 9, 10]
	WES, WGS and RNA‐seq	—	ATM, CHD4, FAT1, KMT2D, MED12, NF2, SUFU, ARID1A, ARID1B, DDR1	Therapeutic target	[11]
Clear cell sarcoma	WES, Sanger sequencing, PCR, direct sequencing.	EWS/ATF1 fusion gene	NBN	Diagnostic markers	[12, 13]
Extra cranial rhabdoid tumor	WGS, whole transcriptome (RNA‐Seq) and miRNA sequencing, ChIP‐seq	—	SMARCB1, CABIN1, SUSD2, SPECC1L and MIF	Therapeutic target	[14, 15, 123, 124, 154, 155, 225, 226
Infantile myofibromatosis	WES, targeted sequencing, allele‐specific PCR, Sanger sequencing and targeted NGS	—	NDRG4, *PDGFRB*	Diagnostic and Prognostic markers.	[16, 17, 18, 19, 20]
Inflammatory myofibroblastic tumor	NGS, FISH, PCR, Sanger sequencing, targeted RNA sequencing	—	*ALK*, *ROS1*, *NTRK*, *RET*, and *PDGFRβ*, JAK1	Therapeutic target	[21, 22, 23, 24, 25]
	IHC assay		*ALK*, *ROS1*	Diagnostic markers	[26]
	NGS		JAK1	Prognostic marker	[27]
Desmoplastic small round cell tumors	WGS, Rt‐PCR and WES	EWSR1‐WT1 translocation		Diagnostic marker	[170, 171, 172]
Leiomyosarcoma	WES, transcriptome sequencing, Sanger, PCR, NGS, WGS, FISH, tissue microarray and IHC assay	—	TP53, RB1, ATRX, RBL2, SP100, ATM, EGFR, PTEN, *MED12*	Diagnostic markers and therapeutic target	[31, 32, 33, 34, 35]
Myxoid/round cell liposarcoma	PCR, FISH, Next‐generation WES, karyotyping and NGS	FUS‐DDIT3 fusion gene	TP53, PIK3CA, RB1 and NF1	Therapeutic target	[36, 37]
Malignant peripheral nerve sheath tumor	WES, NGS, Sanger sequencing, SNP array, PCR, FISH, IHC assay, RNA microarray, karyotype and aCGH	—	*NF1*, *SUZ12*, *EED*, *TP53*, *CDKN2A*, CTNNB1, MED12, CDKN2A/BB, PTEN, TRIM23, NF1, PRC2	Early detection, prognosis markers and therapeutic targets.	[38, 39, 40, 41, 42]
NUT carcinoma	IHC assays, NGS and FISH	BRD4‐NUT fusion gene	*NUTM1*	Diagnostic Markers	[227, 228, 229]
Synovial sarcoma	FISH, PCR, NGS and Sanger sequencing.	Translocation between SSX18 and SSX1, SSX2, or SSX4	*RNF213*, *SEPT9*, *KDR*, *CSMD3*, *MLH1*, *KRAS*, *CCND1 and ERBB4*	Diagnostic marker	[46, 47, 48, 49]

Abbreviations: ALK, Anaplastic Lymphoma Kinase; AP1‐KMT2A, Yes‐Associated Protein 1‐ Lysine Methyltransferase 2A; ARID1A, AT‐Rich Interaction Domain 1A; ARID1B, AT‐Rich Interaction Domain 1B; ATRX, Alpha Thalassemia/Mental Retardation Syndrome X‐Linked; BCB1, SWI/SNF‐Related, Matrix‐Associated, Actin‐Dependent Regulator of Chromatin, Subfamily B, Member 1; CABIN1, Calcineurin Binding Protein 1; CCND1, Cyclin D1; CDKN2A, Cyclin‐Dependent Kinase Inhibitor 2A; CHD4, Chromodomain Helicase DNA Binding Protein 4; CSMD3, CUB and Sushi Multiple Domains 3; DDR1, Discoidin Domain Receptor Tyrosine Kinase 1; EED, Embryonic Ectoderm Development; EWS/ATF1, Ewing Sarcoma Breakpoint Region 1‐Activating Transcription Factor 1; FAT1, Fat Atypical Cadherin 1; FUS, Fused in Sarcoma; JAK1, Janus Kinase 1; KDR, Kinase Insert Domain Receptor; KMT2D, Lysine Methyltransferase 2D; MED12, Mediator Complex Subunit 12; MED12, Mediator Complex Subunit 12; MED12, Mediator Complex Subunit 12; MIF, Macrophage Migration Inhibitory Factor; MLH1, MutL Homolog 1; NBN, Nibrin; NDRG4, N‐Myc Downstream Regulated Gene 4; NF2, Neurofibromin 2; NF2, Neurofibromin 2; NTRK, Neurotrophic Receptor Tyrosine Kinase; NUTM1, Nuclear Protein in Testis Midline Carcinoma Family Member 1; PDGFRB, Platelet‐Derived Growth Factor Receptor Beta; PDGFRβ, Platelet‐Derived Growth Factor Receptor Beta; PIK3CA, Phosphatidylinositol‐4,5‐Bisphosphate 3‐Kinase Catalytic Subunit Alpha; PRC2, Polycomb Repressive Complex 2; PTEN, Phosphatase and Tensin Homolog; PTEN, Phosphatase and Tensin Homolog; RB1, Retinoblastoma Protein 1; RB1, Retinoblastoma Protein 1; RBL2, Retinoblastoma‐Like Protein 2; RET, Rearranged During Transfection Proto‐Oncogene; RNF213, Ring Finger Protein 213; ROS1, ROS Proto‐Oncogene 1, Receptor Tyrosine Kinase; rt‐PCR, real‐time polymerase chain reaction; SEPT9, Septin 9; SH3PXD2A‐HTRA1, SH3 And PX Domains 2A‐ HtrA Serine Peptidase 1; SP100, Nuclear Antigen Sp100; SPECC1L, Sperm Antigen with Calponin Homology and Coiled‐Coil Domains 1 Like; SUFU, Suppressor of Fused Homolog; SUSD2, Sushi Domain Containing 2; SUZ12, Suppressor of Zeste 12 Protein Homolog; TRIM23, Tripartite Motif Containing 23.

### 4.6 Other Rare Tumors

#### 4.6.1 Olfactory Neuroblastoma

Olfactory neuroblastoma (ONB) is a malignant tumor that is localized in the upper portion of the sinonasal tract, and it requires biopsy for a confirmed diagnosis [186]. For treatment, a multimodal approach that includes surgery, chemotherapy, and radiotherapy is required to treat these tumors [186]. Although the exact cause of ONB is unknown, genetic testing of affected individuals reveals alterations within chromosomes 2, 5, 6, 7 and 20 that could be implicated in tumor etiology [187, 188]. WES also identified deletions within the dystrophin (DMD) locus and Laminin Subunit Alpha 2 (LAMA2) in ONB tumors [189, 190]. One study identified seven mutations via WGS and later verified them by Sanger sequencing. Among these mutations are TP53, Thousand and One Kinase 2 (TAOK2), and Mitogen‐Activated Protein Kinase Kinase Kinase Kinase 2 (MAP4K2) genes, which are now known to be the main drivers of carcinogenesis. In case of metastasis, tumors can involve four additional mutations in Kinase Insert Domain Receptor (KDR), Myelocytomatosis Oncogene (MYC), SIN3 Transcription Regulator Family Member B (SIN3B), and NLR Family CARD Domain Containing 4 (NLRC4) [191]. In another study combining Sanger sequencing, gene fusions, whole‐genome RNA microarray, chromogenic and FISH, and IHC, mutations in the TP53, CTNNB1, EGFR, APC, Tyrosine‐Protein Kinase Kit (cKIT), Mesenchymal‐Epithelial Transition Factor (cMET), Platelet‐Derived Growth Factor Receptor Alpha (PDGFRA), Cadherin‐1 (CDH1), FH, and SMAD4 genes were detected in around 63% of analyzed cases. In particular, microarray assays detected the upregulation of Cluster of Differentiation 24 (CD24), Secretogranin‐2 (SCG2), Insulin‐like Growth Factor Binding Protein 2 (IGFBP‐2) genes [192]. TP53 mutation was also identified through NGS and was suggested to be an unfavorable prognostic and predictive factor in ONB [187].

Additional number of tumors should be analyzed to achieve a definitive guide for genetic alterations in ONB in order to meet the increased prevalence of this cancer tumor along with its molecular heterogeneity [193, 194]. Therefore, expanding research collaborations to analyze larger patient cohorts is crucial. Future efforts are required to validate current molecular markers and explore targeted therapy options for ONB.

#### 4.6.2 Thymic Carcinoma

Thymic carcinoma is a malignant epithelial tumor of the thymus characterized by a poor prognosis [195, 196]. This condition is often associated with other thymic tumors, myasthenia gravis, and other autoimmune diseases [197]. Although most cases are treated by surgery, advanced stages necessitate a multimodal treatment approach with radiotherapy and platinum‐based chemotherapy [197]. There is limited information available about the molecular pathology of this disease, which has hindered the development of targeted therapies [198]. Thymic carcinoma can often be misdiagnosed as thymomas, which are a less aggressive type of tumor [199]. Consequently, the use of sequencing technologies such as WES and NGS helped in identifying genetic aberrations that can be used as diagnostic and prognostic markers as well as providing molecular targets to improve the discovery of targeted therapies in thymic carcinoma [198, 200, 201]. For example, the absence of a mutant General Transcription Factor II‐I (GTF2I) oncogene verifies the diagnosis of thymic cancer since these oncogenes are only seen in thymomas [202, 203]. During the early stages of the tumor, using WES, WGS, RNA‐Seq, Sanger sequencing, q‐PCR, or IHC assays can detect mutations in GTF2I, Harvey Rat Sarcoma Virus Oncogene (HRAS), Neuroblastoma RAS Viral Oncogene Homolog (NRAS), and TP53 genes [203, 204]. According to NGS, TP53, KIT Proto‐Oncogene, Receptor Tyrosine Kinase (KIT), and Platelet‐Derived Growth Factor Receptor Alpha (PDGFRA) are the most frequently mutated genes in thymic carcinoma [205, 206]. Survival studies revealed that tumors bearing Receptor Tyrosine Kinase (RTKs) gene mutations in KIT, PDGFRA, or EGFR genes or alterations in the SMAD4 gene have a poor prognosis [205, 207]. Therefore, detailed evaluation of genetic mutations is being achieved through WES or WGS and confirmed by Sanger sequencing [198, 202, 207, 208, 209]. Interestingly, employing the IHC assay along with WES or NGS can further aid in the detection of underlying mutations [199, 210].

To date, targeted therapies for thymic tumors are still showing varied responses across histologic subtypes in addition to their significant toxicities [211]. Multicenter studies with larger cohorts are required to improve understanding and develop effective treatment strategies for this rare malignancy [212]. This can be done by detecting safety biomarkers to improve efficacy and safety, as molecularly directed therapies continue to evolve [212].

## 5 Clinical Translation and Future Perspectives

The implementation of GTST to improve PM in rare cancers requires a directed approach toward clinical implementation strategies, cost‐effectiveness, and patient‐centered outcomes. Over the years, patients with rare cancers have had limited access to scientific evidence needed to establish standard practice guidelines, unlike those with common cancers. Therefore, developing comprehensive guidelines for test evaluation, structuring decision‐making algorithms specific to rare cancers, and using standardized reporting mechanisms are essential for appropriate clinical practice. This can help in detecting genetic alterations responsible for resistance to certain treatments, guiding clinicians in selecting alternative or combination therapies to resolve resistant cases. In terms of cost‐effectiveness, multi‐institutional validation studies should also be conducted to evaluate the affordability and long‐term value of GTST, particularly in healthcare settings with limited resources. This aligns with recent studies emphasizing the need to prioritize GTST use to enhance cost‐effectiveness by reducing reliance on trial‐and‐error treatment approaches and enabling the identification of the most effective therapy from the outset. At a clinical level, patient‐centered outcomes should prioritize improved diagnostic accuracy, personalized treatment plans, and enhanced quality of life, ensuring that the benefits of these technologies directly address patient needs. Recent evidence supports the use of GTST to achieve higher treatment response rates, longer progression‐free survival, and, in certain cases, improved overall survival. Overall, these targeted efforts will facilitate the faster integration of GTST into clinical workflows, advancing PM for rare cancer patients.

## 6 Conclusion

In current practice, the integration of GTST is considered a key component in advancing PM for rare cancers. These approaches have the potential of revealing the genetic complexities of cancer tumors to better understand their intrinsic mechanisms and biological activities. By specifying the underlying genetic alterations driving rare cancer tumors, clinicians can refine treatment strategies to individual patients, maximizing therapeutic efficacy and minimizing adverse effects. The translational impact of these technologies extends beyond diagnosis, guiding treatment selection, therapeutic monitoring, and prognosis assessment. Therefore, the use of GTST is constantly helping scientists to discover diagnostic, therapeutic, and prognostic markers that can drive personalized treatment strategies and improve outcomes for patients with rare cancers. As we continue to harness the power of precision oncology, collaborative efforts among researchers, clinicians, and patients will be essential in optimizing outcomes and improving the quality of care for individuals with rare cancers.

## Author Contributions

B.A.‐O. and J.F. conceptualisation; B.A.‐O. and J.F. methodology; B.A.‐O. and J.F. figures ideas and design; J.F. and L.A. writing – original draft; J.F., L.A., M.A., A.A., and B.A.‐O. writing – review and editing; B.A.‐O. supervision; B.A.‐O. and J.F. project administration. All authors have read and agreed on the final version of the manuscript.

## Ethics Statement

The authors have nothing to report.

## Conflicts of Interest

The authors declare no conflicts of interest.

## Data Availability

Data sharing is not applicable to this article as no new data were created or analyzed in this study.
